# Cerebral perfusion pressure targets after traumatic brain injury: a reappraisal

**DOI:** 10.1186/s13054-025-05458-9

**Published:** 2025-05-21

**Authors:** Stefan Yu Bögli, Ihsane Olakorede, Erta Beqiri, Xuhang Chen, Andrea Lavinio, Peter Hutchinson, Peter Smielewski

**Affiliations:** 1 Brain Physics Laboratory, Division of Neurosurgery, Department of Clinical Neurosciences, University of Cambridge, Cambridge, UK; 2 Division of Neurosurgery, Department of Clinical Neurosciences, University of Cambridge, Cambridge, UK; 3 Division of Anaesthesia, Department of Medicine, University of Cambridge, Cambridge, UK; 4 Department of Neurology and Neurocritical Care Unit, Clinical Neuroscience Center, University Hospital Zurich, University of Zurich, Zurich, Switzerland

**Keywords:** Traumatic brain injury, Multimodality monitoring, Cerebral perfusion pressure, Cerebrovascular autoregulation, Blood pressure targets

## Abstract

**Introduction:**

Cerebral perfusion pressure (CPP) management is central to neurocritical care management after traumatic brain injury (TBI). While the Brain Trauma Foundation recommends a target of 60–70 mmHg, it is unclear whether this range reflects the lower limit or the optimal level, when viewed through the prism of cerebrovascular autoregulation. Autoregulation aims at stabilizing cerebral blood flow and can be estimated continuously using the pressure reactivity index (PRx). Personalized CPP targets can be derived from PRx including CPPopt (optimal CPP), LLA, and ULA (lower and upper limit of autoregulation). Emerging data suggests an asymmetric relationship around CPPopt, with more pronounced autoregulation deterioration with decreasing compared to increasing CPP. Based on the hypothesis that higher CPP levels may be less harmful than lower CPP levels, we aimed to reassess rigorously the prognostic value of the different CPP targets.

**Material and methods:**

We analyzed 809 adult TBI patients admitted from 2002 to 2023 who underwent invasive intracranial pressure monitoring and had available 6-month outcomes. CPPopt, LLA, and ULA were estimated using previously published methodologies. Deviations of CPP from fixed or personalized targets were assessed describing the overall dose, the hourly dose and the percentage time spent outside of these targets and examined using Chi-squared tests, logistic regressions, heatmaps, ordinal analyses, and group-based trajectory modelling.

**Results:**

Our data confirms an asymmetric CPP/PRx relationship with steeper increases in PRx with decreasing and only modest elevations with increasing CPP. Even small decreases below CPPopt were consistently linked to worse outcomes (OR 1.04 (CI 1.02–1.06) and 1.09 (CI 1.04–1.15) for hourly dose and percentage time spent below CPPopt, *p* < 0.001 and *p* = 0.001 respectively). The strength of association increased with further decreases in CPP away from CPPopt towards the LLA with OR of 1.11 (CI 1.07–1.14) and 1.26 (CI 1.18–1.35) for hourly dose and percentage time spent below LLA respectively (*p* < 0.001). Conversely, higher-than CPPopt levels generally showed no association to worse outcomes. Distinct trajectories in the relationship between CPPopt and LLA (introduced as the Lower Limit Margin) could be identified with worse outcomes in those with decreasing distance between these targets (Mortality of 18% vs. 45% for patients with increasing vs. decreasing lower limit margins, *p* = 0.003).

**Conclusion:**

Our findings corroborate experimental work suggesting that TBI patients are more vulnerable to CPP reductions below as compared to elevations above personalized thresholds. The results highlight the need for individualized CPP management strategies that prioritize avoidance of lower CPP levels and suggest using CPPopt as the lower CPP limit.

**Supplementary Information:**

The online version contains supplementary material available at 10.1186/s13054-025-05458-9.

## Introduction

Cerebral perfusion pressure (CPP) represents one of the key measures during intensive care management after traumatic brain Injury (TBI). The Brain Trauma Foundation suggests targets ranging from 60 to 70 mmHg but emphasizes that it remains unclear whether these represent the lower limit or the optimal target [1]. Cerebrovascular autoregulation represents a mechanism which supports stabilizing cerebral blood flow in response to changes in systemic driving pressure by adjusting arteriolar diameter and thereby vascular resistance [2]. This mechanism is intact within patient specific ranges [3]. Outside of this range, i.e. beyond the bounds of the lower or upper limit of autoregulation (LLA and ULA respectively), cerebral blood flow is not stabilized sufficiently leading to the risk of hypoxia or hyperperfusion, respectively [4]. In intensive care, the pressure reactivity index PRx represents the most commonly applied continuous cerebrovascular autoregulation proxy measure [5]. PRx quantifies the change in intracranial pressure (ICP) to changes in arterial blood pressure and is consequently thought to reflect the character of changes in arteriolar diameter. In 2002, the U-shaped relationship between CPP and PRx was first described, giving rise to the concept of optimal CPP (i.e. CPPopt), [6] which reflects the CPP associated with the lowest PRx across the different CPP levels. Similarly, LLA and ULA can be derived from this curve by extracting the CPP value at which cerebrovascular reactivity is already impaired and further decreases (for LLA) or increases (for ULA) in CPP further compromise reactivity. While deviations from CPPopt and LLA have consistently been associated to outcomes [6–9], deviations from ULA remain understudied with somewhat unclear results [7, 8].

The currently available automated methods [7, 10, 11] used for the estimation of the relationship assume a bilaterally symmetric U-shaped relationship and the region representing optimal reactivity has relatively arbitrarily been defined as within 5 mmHg above or below CPPopt. However, experimental findings in animals by Klein et al. [12] challenge these assumptions. By directly visualizing changes in pial arteriolar blood flow in response to induced alterations in CPP, they reproduced the complete autoregulation curve. Notably, while the breakpoint associated with the LLA exhibited the anticipated pattern—a single, well-defined breakpoint—the upper portion demonstrated a much more gradual transition from preserved to impaired cerebrovascular autoregulation. Specifically, with increasing CPP, there was initially a very modest rise in cerebral blood flow (attributed to the failure of small arterioles to constrict) followed by a progressively steeper increase in flow, reflecting the eventual overpowering of the arteriolar smooth muscle in larger vessels. In contrast, passive flow responses below the LLA occurred when further arteriolar dilation was insufficient to reduce cerebrovascular resistance enough to sustain adequate cerebral perfusion [13].

Cerebral hypoperfusion is widely, and largely unequivocally, recognized as a critical determinant of poor outcome following TBI. Conversely, the potential consequences of cerebral hyperperfusion with CPP beyond the ULA, remain relatively underexplored, even though it may lead to secondary injury through mechanisms such as blood–brain barrier disruption, cerebral edema, and hemorrhagic transformation, particularly after the sustained brain injury [2, 14]. Building on the experimental findings by Klein et al., which suggest a more gradual loss of autoregulatory function at higher CPPs, our study aims to rigorously re-examine the CPP/PRx relationship, explicitly testing the hypothesis that this curve is indeed asymmetric and consequently increasing CPP beyond CPPopt may not be as detrimental to the brain’s physiology compared to decreases in CPP below CPPopt. By doing so, we seek to refine the characterization of derived CPP targets (CPPopt, LLA, and ULA), and to assess their relative prognostic value. From a clinical perspective, this investigation seeks to clarify the safety margin for increasing CPP beyond CPPopt or the LLA without adversely affecting outcomes following TBI.

## Materials and methods

The acquisition and use of this dataset was approved by the local ethics committee (REC 23/YH/0085). The dataset includes high-resolution monitoring data and clinical descriptors from consecutive TBI patients admitted to the Neurocritical Care Unit Addenbrooke’s Hospital, Cambridge University Hospital NHS Foundation Trust, University of Cambridge. The patients continuously receive monitoring for the duration of their stay if deemed necessary (i.e. monitoring is stopped if the patient receives redirection of care to palliative measures or if the patient has been sufficiently stabilized, is clinically assessable, and no further secondary injuries are foreseeable). All data was obtained as part of routine care. No study-specific data was collected, and therefore, the requirement for informed consent was waived.

The management of the patients largely follows the Brain Trauma Foundation guidelines [1]. The adapted local guidelines have previously been described [15] and the ICP/CPP treatment protocol can be found in Supplement A. The changes in management throughout the recruitment period have previously been described [16]. PRx and CPPopt were introduced at the bedside in 1999 and 2012 respectively. While targeting CPPopt is part of the advised Tier 1 ICP/CPP management, it is still up to the treating physician whether such target should be followed. Of note, while such management can, under certain circumstances, improve cerebrovascular reactivity as assessed by PRx [17], no currently available method augments the shape of the underlying CPP/PRx relationship.

### Study population

Consecutive patients admitted between 01.2002 and 12.2023 were evaluated for inclusion. Inclusion criteria were: 1. Acute TBI with invasive ICP monitoring; 2. Available 6-month outcome (Glasgow Outcome Scale–GOS). There were no specific exclusion criteria. Between 2002 and 2020 the decision for data acquisition was dependent on the availability of research staff. A systematic acquisition was started thereafter, as described in previously published flowcharts [18]. The following clinical data was extracted from the database: sex, age, glasgow coma scale (GCS), pupillary reactivity (both reactive vs. one reactive vs. none reactive), presence of intracranial bleed (extradural, intracerebral, or subdural hematoma, contusion, traumatic subarachnoid hemorrhage), presence of extracerebral injuries (divided into injuries to thorax, abdomen, extremities, pelvic, skull, or spine), presence of isolated TBI (no extracranial injuries), decompressive craniectomy (DC), and GOS. GOS was assessed 6 months after ictus during outpatient consultations or via telephone interviews by trained staff. Outcome was evaluated assessing the ordinal scale (good recovery vs. moderate disability vs. severe disability vs. dead/vegetative) or as dichotomized outcome (GOS 1–3 vs. 4–5).

### Monitoring data acquisition and preprocessing

High resolution (i.e. waveform, 100–240 Hz) physiological data was collected in real time at the bedside from GE Solar and subsequently Carescape B650monitors (General Electrics, Massachusetts, USA), and pre-processed, using the ICM + software [19] (Cambridge Enterprises, University of Cambridge, UK). ICP was measured using intraparenchymal pressure transducers (Codman ICP MicroSensor, Codman & Shurtleff, Raynham, Massachusetts). ABP was measured using arterial lines (Baxter Healthcare, Deerfield, Illinois, USA) inserted into the radial or femoral artery and zeroed at the level of the foramen of Monro (after 2015, as estimated by tragus level) or at the level of the right atrium (before 2015) [16]. To account for these differences, an offset of 10 mmHg was deducted from the ABP readings in patients studied prior to 2015, based on the standard management protocol (which includes 30° head elevation [15]) and the associated difference in hydrostatic pressure [20]. Raw ABP and ICP signals were curated as previously described [18] to remove the following artifacts: Sections with arterial line failure, readings outside of physiological ranges or without a pulse, and readings of artefactual nature (high spectral edge frequency). The resulting artifact free data was then processed to acquire 10 s averages of ABP, ICP, and CPP (difference between average ABP and ICP). PRx [5] was calculated by computing the moving correlation coefficient between 10-s mean ABP and ICP values over consecutive 5-min intervals. CPPopt, LLA, and ULA were estimated automatically every minute for consecutive 8 h data windows as described previously by fitting a parabolic curve to 5-min median CPP and PRx values using the multi-window weighted approach [7, 10]. The PRx cutoff for LLA and ULA was chosen to be 0.3. The lower limit margin was calculated as the difference between CPPopt and LLA. For the subsequent analyses, minute-by-minute averages were used.

### Statistical analysis and visual exploration

Statistical analyses and figure preparation were performed using R studio software (version 4.4.1; the specific packages used are described in Supplement B). Statistical significance was set at the level of 0.05.

The relationship between CPP and PRx was initially examined using visual methods, including histograms and boxplots, based on both the complete dataset and subsets stratified by outcome (favorable vs unfavorable), ICP (high vs. low—i.e. above vs. below 20 mmHg), and age group. Specifically, for each patient, the average PRx was computed for each 5 mmHg CPP bin spanning from 30 to 150 mmHg, and the corresponding count of CPP observations was recorded to allow for assessing the proportion of data within the respective bins. These individual values were subsequently aggregated by averaging for visualization purposes and bins with less than 10 patients being removed.

For statistical analysis, deviations from LLA, CPPopt, ULA and a series of fixed CPP targets (between 30 and 120 mmHg) were explored. For each of these CPP targets we calculated the following three metrics considering either the section above (for ULA, CPPopt, and fixed CPP thresholds above 65 mmHg) or below (for LLA, CPPopt, and fixed CPP thresholds below 65 mmHg): (1) Dose, defined as the area under the curve above or below the cutoff, respectively; (2) hourly dose (hDose), representing the total dose divided by hours of valid data; and (3) percentage time (ptime), of valid data, during which CPP was above or below the target. Notably, hDose and ptime are less influenced by total monitoring duration, as they reflect deviations relative to the available monitoring time, whereas the absolute dose tends to increase with longer monitoring periods.

Two univariable methods were employed to extrapolate the association between different CPP cutoffs and outcomes. First, stepwise Chi-squared tests [21] were employed to assess the association between different CPP or PRx cutoffs (in steps of 1 mmHg or 0.05, respectively) and outcome. Second, logistic regression models were used to explore the magnitude of association between CPP Dose, hDose, and ptime at different levels and dichotomized outcomes (GOS 1–3 vs. 4–5). For data acquired prior to 2021 only limited clinical information is available, consequently multivariable analyses were performed using the newer dataset only. Specifically, to account for differences in clinical severity, prognostic scores were used to match patients. Briefly, patients with favorable and unfavorable outcome were matched based on prognostic risk scores (estimated using logistic regression using the variables age, sex, motor GCS, pupillary reactivity, type of hemorrhage, isolated TBI vs. polytrauma, extracranial injury (to the abdomen, extremities, pelvis, skull, spine, or thorax separately), and DC) to acquire a cohort with similar clinical presentation but differing outcomes. Matching was performed using the nearest-neighbor method with a caliper of 0.2 and 1:1 matching. Matching quality was verified visually (density and point distribution plots) and quantitatively (statistical analysis). The association between the described CPP targets and outcome were then assessed using Kruskal–Wallis rank sum tests as appropriate.

For exploiting the granular data, different visualizations based on the initial description by Guiza [22] were produced by adapting a publicly available code [23]. Three different heatmaps were produced. First, to explore the association between magnitude and duration of CPP deviations relative to LLA, ULA, or CPPopt and outcome, minute-by-minute deviations of CPP from these targets were explored. For each patient, a grid was constructed to depict the frequency of various combinations of minimum deviation and minimum duration. The minimum deviation values were defined for LLA and ULA over a range of –30 to 30 mmHg and for CPPopt over –40 to 40 mmHg, with each cell representing a 2 mmHg increment. Similarly, the minimum duration values ranged from 5 to 120 min, with 2-min increments per cell. For deviations below the LLA threshold and for CPP values below CPPopt, the minimum deviation and duration were estimated based on the extent to which CPP fell below the designated cutoffs. For deviations above the ULA threshold and for CPP values above CPPopt, the minimum deviation and duration of CPP exceeding these cutoffs were used to define the insult. To create the heatmaps, for each cell, weighted Pearson correlation (using the number of patients as weights) was used to assess the relationship between the average number of occurrences or percentage time per outcome category. The resulting maps were then visualized using a color map ranging from red to blue representing associations to worse and better outcome respectively. To improve visual interpretability, cells were smoothed as previously described [24, 25], where each cell of the grid was divided into 3*3 smaller cells and this was followed by application of a Gaussian kernel filter (standard deviation of 2 pixels). Grid cells with less than 40 patients with the specific cell combination were colored grey.

Considering the ordinal nature of GOS we additionally performed two ordinal analyses, namely proportional odds logistic regression and a sliding dichotomy analysis [26]. To increase statistical power, proportional odds logistic regression provides a common odds ratio reflecting the average shift over the full ordinal scale, while sliding dichotomy utilizes baseline adjusted outcome definitions thereby identifying whether patients do worse than expected [26]. Proportional odds logistic regression was applied to the GOS scale with moving cutoffs (Dead/Vegetative vs. Severe Disability, Severe Disability vs. Moderate Disability, Moderate Disability vs. Good Recovery) to assess the common odds ratio. The proportional odds assumption was not formally tested, as the primary aim was to assess the overall direction and magnitude of association across the ordinal outcome scale rather than providing evidence that this change is the same for each cutoff. Notably, the common odds ratio derived from the model still provides a meaningful summary of the effect, even when the proportional odds assumption is not strictly met [27]. Lastly a sliding dichotomy approach was employed to assess the importance of CPP deviations for a baseline severity adjusted outcome definition. For each patient, based on the baseline covariates (age, sex, motor GCS, pupillary reactivity, type of hemorrhage, isolated TBI vs. polytrauma, extracranial injury (to the abdomen, extremities, pelvis, skull, spine, or thorax separately), and DC), a prognostic risk probability for unfavorable outcome was estimated. The resulting scores were then divided into 3 prognostic groups of roughly equal size corresponding to low, intermediate, and high likelihood of unfavorable outcome. For each prognostic group a separate cutoff was defined to dichotomize outcome into favorable and unfavorable, with the adjusted favorable outcome classified as: GOS 5 for the group with low likelihood for unfavorable outcome, GOS 4–5 for the group with intermediate likelihood for unfavorable outcome, and GOS 3–5 for the group with high likelihood for unfavorable outcome. The resulting baseline severity adjusted outcome variable was then assessed against the CPP metrics using logistic regression. For both methods bootstrapping was applied for internal validation and to acquire the 95% confidence interval (CI).

Lastly, based on previously described methods [28–30], group based trajectory modeling was applied to identify subgroups of individuals with similar lower limit margin trajectories (assessed as 6 hourly non-overlapping averages) within the first 7 days of monitoring in the cohort acquired after 2021 with available exact ictus time and date. The lower limit margin was specifically chosen since it is independent of the absolute value of either CPPopt or LLA and therefore directly comparable between patients. For this purpose, the optimal number of trajectory subgroups (exploring 2 to 4 trajectories) was selected using the Bayesian Information Criterion. For the optimal number of trajectories identified, the effect of decreasing the polynomial degree was explored (exploring 1 to 4 degrees). The model with the optimal polynomial degree was then chosen for further analysis. To assess model adequacy, at each step, the average posterior probability of assignment was used [31]. Lastly, each patient was assigned to the respective trajectory group, and the corresponding clinical information was then compared using Kruskal–Wallis rank sum tests.

## Results

A total of 809 patients requiring invasive neuromonitoring after TBI were included in this study. Their clinical parameters, stratified by acquisition year, are summarized in Table 1. Of these patients, 77% were male, with a median age of 45 years (IQR 25–55) and an initial GCS motor score of 4 (IQR 1–5). During hospitalization, 31% underwent DC. At 6 months post-injury, 27% of patients had died or remained in a vegetative state, while 46% achieved a favorable outcome (i.e. Good Recovery or Moderate Disability). Overall, 150′756 h of monitoring data were assessed, corresponding to a median of 163 h (IQR 75–249) per patient. The distribution and temporal coverage of CPP stratified by outcome category are detailed in Supplement C. No differences in monitoring length depending on outcome category could be identified. The median yield of CPPopt during the overall monitoring period was 79.7% (IQR 67.1–86.1).

**Table 1:** Patient characteristics*

Characteristic	Cohort		
	Total	TBI 2002–2020	TBI 2021–2023
	*N* = 809	*N* = 575†	*N* = 234
Sex (male)	567 (77%)	391 (78%)	176 (75%)
Age (years)	45 (25, 55)	40 (25, 55)	45 (35, 65)
Motor GCS	4 (1, 5)	4 (2, 5)	4 (1, 5)
*Pupillary reactivity*			
Both reactive	392 (76%)	235 (84%)	157 (67%)
One reactive	72 (15%)	36 (13%)	36 (15%)
None reactive	51 (9.5%)	10 (3.6%)	41 (18%)
DC	230 (31%)	160 (31%)	70 (30%)
Isolated TBI	NA	NA	54 (23%)
*Intracranial injury*			
EDH	NA	NA	33 (14%)
ICH	NA	NA	48 (21%)
SDH	NA	NA	160 (68%)
Contusion	NA	NA	140 (60%)
tSAH	NA	NA	154 (66%)
*Extracranial injury*			
Abdomen	NA	NA	34 (15%)
Bone extremities	NA	NA	43 (18%)
Pelvis	NA	NA	26 (11%)
Skull	NA	NA	128 (55%)
Spine	NA	NA	49 (21%)
Thorax	NA	NA	100 (43%)
*GOS*			
Good recovery	146 (18%)	115 (20%)	31 (13%)
Moderate disability	230 (28%)	163 (28%)	67 (29%)
Severe disability	212 (26%)	147 (26%)	65 (28%)
Dead/vegetative	221 (27%)	150 (26%)	71 (30%)

Various patient characteristics are presented for the cohorts acquired (TBI 2002–2020 and TBI 2021–2023). Within the initial cohort, only basic clinical parameters were acquired, while the latter cohort included relevant prognostic parameters. When comparing the different TBI cohorts, no differences could be identified for patient sex, initial motor GCS, frequency of DC, and overall distribution of GOS (*p* > 0.05). When assessing GOS within subgroup analyses, patients within the cohort TBI 2021–2023 were less likely to achieve good recovery, *p* = 0.024. Additionally, patients admitted as part of the TBI 2021–2023 were older (*p* < 0.001) and more likely presented with non-reactive pupils (*p* < 0.001)

*GCS* glasgow coma scale, *GOS* glasgow outcome scale, *DC* decompressive craniectomy, *EDH* extradural hematoma, *ICH* intracerebral hematoma, *NA* not available, *SDH* subdural hematoma, *tSAH* traumatic subarachnoid hemorrhage, *TBI* traumatic brain injury

^†^Missing data: Sex (13%), Age (0.5%), Motor GCS (57%), Pupillary reactivity (51%), DC (12%)

^*^Data shown as median (interquartile range) or number (%)

**Fig. 1:**
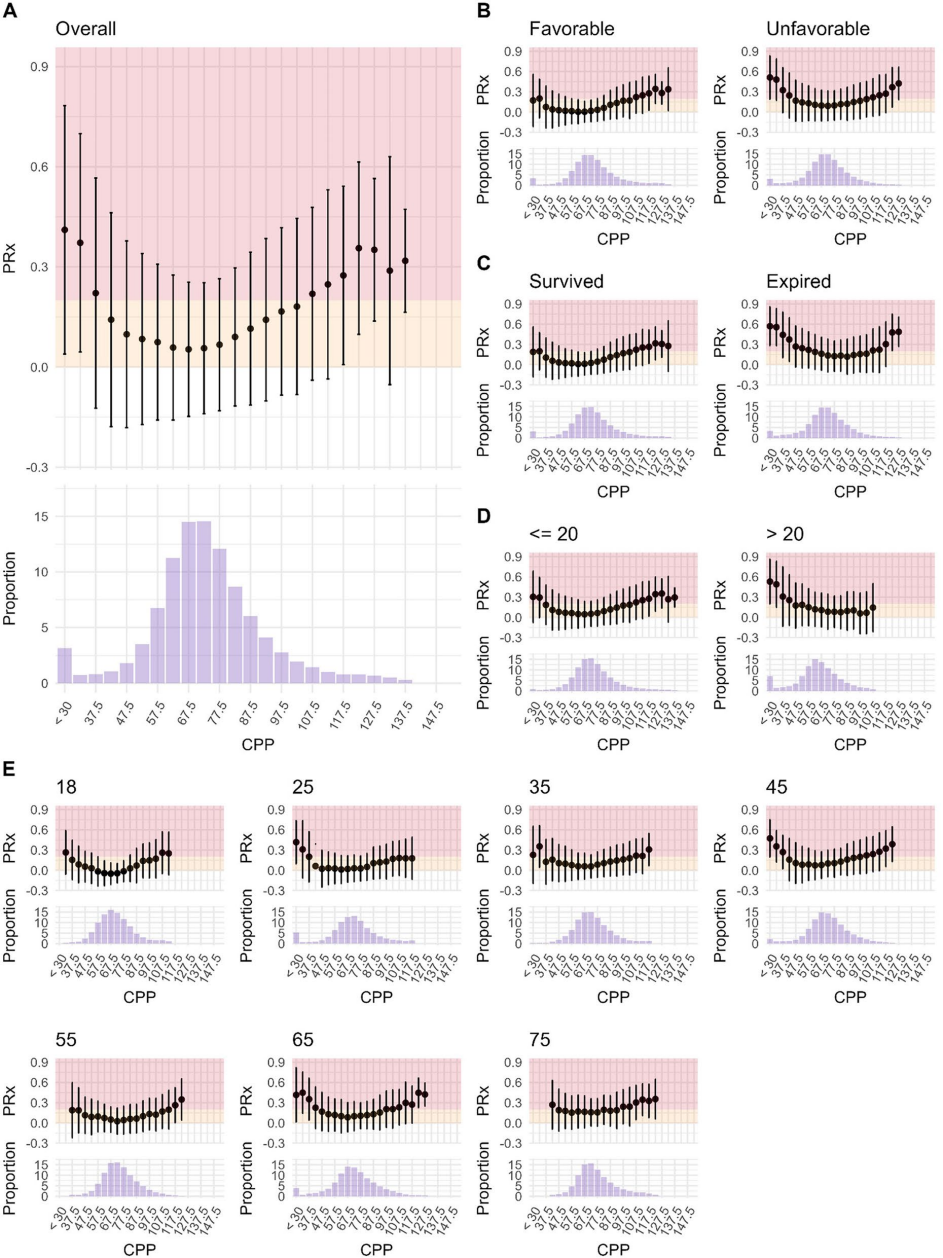
The relationship between CPP and PRx. The following figures were prepared including the full patient cohort (*N* = 809). The relationship between CPP and PRx are displayed considering all patients (panel A) or stratified by outcome (panel B), mortality (panel C), level of ICP (panel D, considering above vs below 20 mmHg), or age (panel E). For the age group 18, all patients aged below 20 years were considered. For all the other age groups, the age described + / − 5 years were considered (i.e. for 25 years, patients aged 20 to 29 years were included)

Figure 1 depicts the various CPP/PRx relationships. When considering the full dataset (Fig. 1A), PRx rises sharply with CPP values below approximately 50 mmHg. In contrast, the increase in PRx is more gradual for increasing CPP values, with PRx exceeding 0.3 around roughly 110 mmHg. When stratifying the data by outcome (Fig. 1B) or mortality (Fig. 1C) the LLA was higher in non-survivors or patients with unfavorable outcomes, while the ULA remained around 110 mmHg regardless of outcome. Although the general pattern held across different age groups (Fig. 1E), the lowest PRx achieved at the optimal level (PRxopt) increased with increasing age, resulting in a flattening of the relationship ultimately remaining above a value of 0 for all CPP bins in the oldest age group. Lastly, patients with high ICP (Fig. 1D) appeared to reach both LLA and ULA at higher CPP values compared to patients with low ICP.

**Fig. 2:**
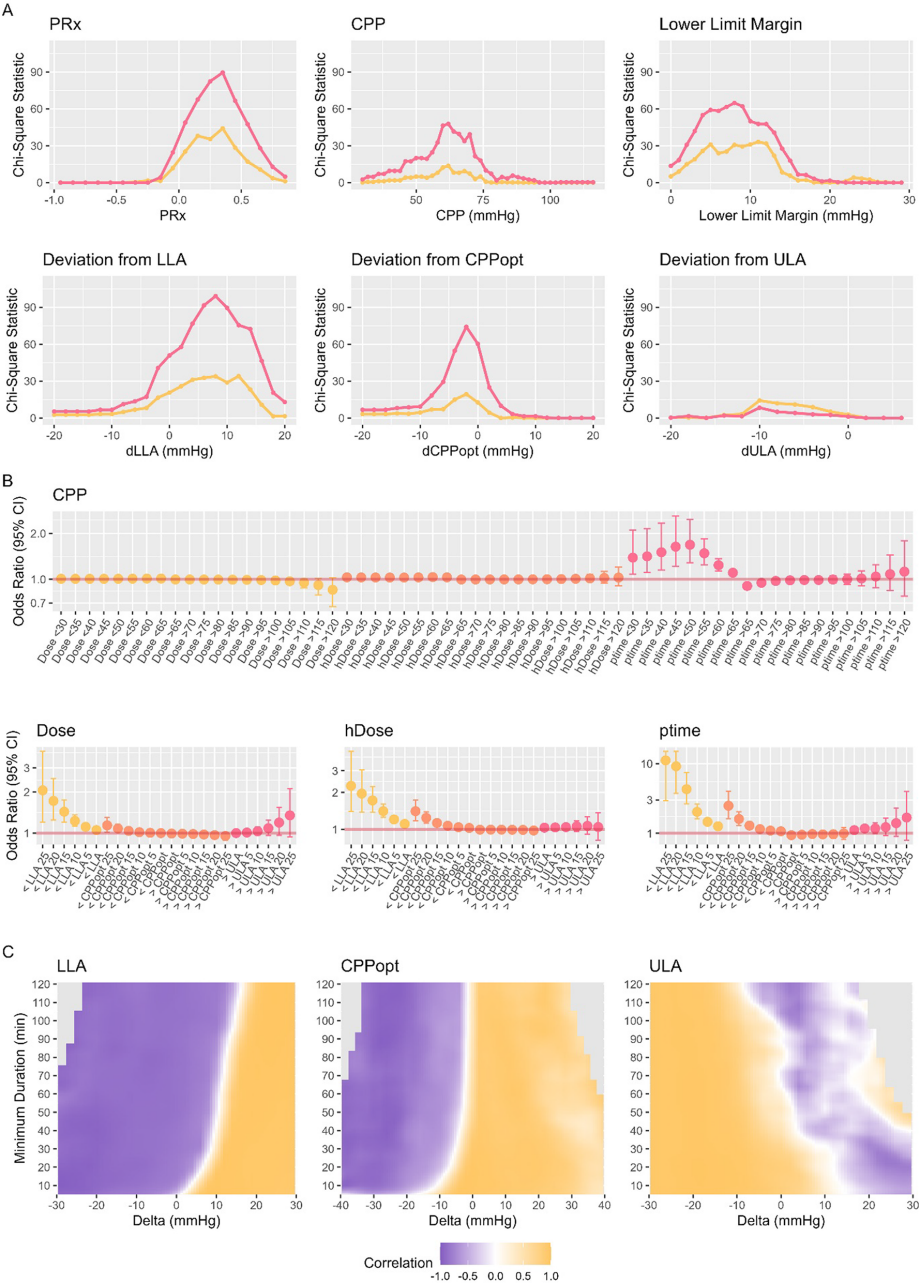
The association between CPP targets and outcome: Chi-squared tests, logistic regression, heatmaps. The following figures were prepared including the full patient cohort (N = 809). Panel A displays the results of the Chi-squared testing for PRx, CPP, and the autoregulation derived measures. The results are displayed either for the differentiation of mortality (red) or outcome (yellow). Panel B displays the results of the logistic regression analyses for the different raw or autoregulation derived CPP targets considering overall dose (Dose); hourly dose (hDose); or percentage time (ptime). The derived odds ratios are displayed for every 1000 mmHg*h Dose, 10 mmHg hDose, and 5% ptime. For the personalized CPP targets, the number behind the target represents the deviation from the cutoff (e.g. LLA 5 uses the cutoff that is 5 mmHg below the LLA). The corresponding OR and p-values are shown in Supplement D. Panel C displays the heatmaps describing the associations between deviations from LLA (left), CPPopt (middle), ULA (right) and outcome. The associations to worse outcome are colored blue, while the associations to better outcome are colored yellow with the depth corresponding to the strength of association. The most distinct increase in association can be appreciated with any time spent below the LLA. Even if sufficient time is spent close, but still above, the LLA, there is an association with worse outcome. Similarly, for CPPopt, time spent below the optimum is associated with unfavorable outcome while there seems to be a distinct safe band above the CPPopt. For ULA, only prolonged deviations above the limit were weakly associated with worse outcomes. The key aspect to appreciate in these heatmaps is the white transition zone, which indicates where the association shifts from favorable to unfavorable outcomes, as well as the consistency of this association. The strength of these associations is quantified in the formal statistical analyses

Figure 2A presents the findings of the Chi-squared testing. A PRx threshold of 0.3 emerged as optimal for identifying mortality, while the threshold for distinguishing favorable from unfavorable outcomes were between 0.2 and 0.3, with the highest Chi-squared statistic occurring at 0.3. For average CPP values, the optimal cutoff was approximately 60 mmHg. When assessing deviations from CPPopt, average CPP values below CPPopt were identified as optimal for differentiating outcome. Moreover, as CPP values approached either the LLA or ULA, their ability to discriminate outcomes increased—most notably when nearing the LLA. These findings were confirmed through logistic regression analyses (Fig. 2B and Supplement D). In general, deviations from fixed CPP were weak predictors of unfavorable outcomes, except for ptime below 60 mmHg. In contrast, deviations from personalized CPP targets proved to be more predictive, particularly for ptime, Dose, or hDose values below LLA. While deviations below CPPopt were also associated with worse outcomes, no similar relationship was observed for deviations above CPPopt; indeed, on the whole, higher values above CPPopt were associated with better outcomes up to the ULA, with deviations extending beyond ULA displaying a trend toward poorer outcomes, these results did, however, not reach statistical significance. The exact p-values and odds ratios for the different cutoffs are presented in Supplement D. Figure 2C illustrates the heatmaps. The analysis revealed that the most pronounced association with unfavorable outcomes occurred when CPP fell below the LLA. Notably, even when CPP remained above the LLA, prolonged exposure at levels up to 10–20 mmHg above this threshold were still linked to worse outcomes. This observation was further supported by the CPPopt heatmap, which indicates that both the duration and magnitude of time spent below CPPopt were increasingly associated with adverse outcomes. Conversely, a distinct safe zone appeared to exist above CPPopt, extending up to the ULA, where outcomes tended to be more favorable unless the ULA was crossed for an extended period or higher magnitude, although the associations here were much weaker and inconsistent compared to the LLA case.

Table 2 presents the statistical analyses for patients matched using prognostic scores (the matching procedure is described in Supplement E), which align with univariable results. The results of the ordinal analyses are described in Table 3. Overall, when assessed using the sliding dichotomy approach, the strongest associations were identified for CPP below the LLA and a fixed lower CPP threshold of 60 mmHg followed by CPP below CPPopt. While these associations could also be identified within the proportional odds regression analyses, there was an additional moderate association between CPP above the ULA and worse outcomes.

**Table 2:** Different CPP derived metrics stratified by outcome category after matching based on prognostic risk scores*

	Characteristic	Good recovery *N* = 16	Moderate disability *N* = 46	Severe disability *N* = 37	Dead/vegetative *N* = 25	*p*-value
CPP	Average (mmHg)	72 (70, 77)	75 (71, 79)	75 (73, 78)	73 (71, 77)	0.2
	Dose < 60 (mmHg*h)†	2.18 (0.66, 3.24)	1.00 (0.39, 1.81)	1.72 (0.34, 3.81)	1.57 (0.85, 5.18)	0.10
	Dose > 70 (mmHg*h)†	52.8 (12.0, 189.3)	56.1 (26.4, 133.3)	62.9 (29.3, 149.3)	37.8 (19.1, 93.9)	0.5
	hDose < 60 (mmHg)	9 (6, 15)	8 (3, 14)	9 (2, 18)	15 (6, 25)	0.051
	hDose > 70 (mmHg)	291 (194, 513)	380 (217, 630)	412 (322, 677)	282 (192, 462)	0.081
	ptime < 60 (%)	3.0 (2.4, 7.5)	3.0 (1.3, 6.8)	3.0 (1.0, 6.5)	5.8 (1.9, 9.7)	0.14
	ptime > 70 (%)	62 (48, 76)	70 (56, 80)	67 (59, 84)	61 (52, 73)	0.12
CPPopt	Average (mmHg)	72 (69, 79)	74 (71, 77)	74 (70, 77)	74 (70, 78)	0.9
	Dose < -5 (mmHg*h)†	11.9 (3.1, 23.5)	9.8 (5.2, 22.5)	10.31 (2.5, 22.9)	13.8 (6.1, 25.5)	0.7
	Dose > + 5 (mmHg*h)†	20.9 (3.6, 37.5)	16.6 (10.1, 31.3)	25.28 (7.6, 38.7)	10.9 (2.3, 19.8)	0.073
	hDose < -5 (mmHg)	78 (67, 107)	94 (55, 114)	75 (38, 135)	137 (99, 178)	0.008
	hDose > + 5 (mmHg)	122 (81, 160)	131 (89, 173)	156 (117, 213)	74 (58, 126)	0.002
	ptime < -5 (%)	25 (22, 28)	26 (19, 30)	21 (15, 30)	36 (25, 42)	0.001
	ptime > + 5 (%)	35 (25, 36)	34 (27, 39)	36 (30, 44)	24 (15, 30)	< 0.001
LLA	Average (mmHg)	57 (55, 62)	59 (56, 64)	58 (54, 64)	61 (58, 64)	0.3
	Dose < 0 (mmHg*h)†	1.7 (1.1, 4.3)	1.9 (0.6, 5.7)	3.0 (0.6, 4.6)	9.4 (2.9, 15.5)	0.021
	hDose < 0 (mmHg)	11 (7, 25)	18 (6, 48)	19 (4, 45)	74 (32, 198)	0.002
	ptime < 0 (%)	4 (3, 9)	7 (4, 13)	5 (2, 10)	17 (10, 46)	0.001
ULA	Average (mmHg)	89 (86, 94)	90 (85, 94)	91 (85, 96)	88 (79, 95)	0.5
	Dose > 0 (mmHg*h)†	3.5 (1.6, 7.0)	4.4 (1.7, 9.8)	3.4 (1.7, 10.5)	3.5 (1.6, 7.2)	0.9
	hDose > 0 (mmHg)	23 (6, 59)	37 (20, 67)	33 (9, 71)	34 (14, 67)	0.6
	ptime > 0 (%)	7 (3, 13)	10 (6, 16)	9 (4, 17)	11 (5, 21)	0.4

*CPP* cerebral perfusion pressure, *CPPopt* optimal CPP, *hDose* hourly dose, *LLA* lower limit of autoregulation, *ULA* upper limit of autoregulation, *ptime* percentage time

^*^Data shown as median (interquartile range); †shown in 1000 mmHg*

**Table 3:** Ordinal analyses*

	Characteristic	Sliding dichotomy	Proportional odds regression
		OR (95% CI)	OR (95% CI)
CPP	Average (mmHg)	0.97 (0.94–1.01)	0.98 (0.95–1.01)
	Dose < 60 (mmHg*h)	**1.13 (1.03–1.26)**	**1.07 (1.02–1.16)**
	Dose > 70 (mmHg*h)	1.00 (1.00–1.00)	1.00 (0.99–1.00)
	hDose < 60 (mmHg)	**1.17 (1.02–1.43)**	**1.13 (1.03–1.30)**
	hDose > 70 (mmHg)	1.00 (0.99–1.00)	1.00 (0.99–1.01)
	ptime < 60 (%)	**1.20 (1.02–1.54)**	**1.24 (1.07–1.50)**
	ptime > 70 (%)	0.98 (0.94–1.05)	0.94 (0.88–1.01)
CPPopt	Average (mmHg)	0.98 (0.94–1.03)	1.00 (0.96–1.04)
	Dose < -5 (mmHg*h)	**1.02 (1.00–1.04)**	1.01 (0.99–1.02)
	Dose > +5 (mmHg*h)	1.00 (0.99–1.01)	0.99 (0.98–1.00)
	hDose < -5 (mmHg)	**1.05 (1.01–1.09)**	**1.06 (1.02–1.10)**
	hDose > +5 (mmHg)	0.99 (0.95–1.02)	1.00 (0.97–1.03)
	ptime < -5 (%)	1.12 (0.99–1.27)	**1.17 (1.05–1.31)**
	ptime > +5 (%)	0.91 (0.80–1.02)	0.92 (0.83–1.02)
LLA	Average (mmHg)	1.00 (0.96–1.04)	**1.04 (1.01–1.08)**
	Dose < 0 (mmHg*h)	**1.05 (1.01–1.09)**	**1.06 (1.03–1.10)**
	hDose < 0 (mmHg)	**1.09 (1.03–1.15)**	**1.15 (1.09–1.22)**
	ptime < 0 (%)	**1.17 (1.04–1.34)**	**1.36 (1.22–1.53)**
ULA	Average (mmHg)	0.98 (0.95–1.01)	0.96 (0.94–0.99)
	Dose > 0 (mmHg*h)	1.01 (0.97–1.05)	1.00 (0.97–1.04)
	hDose > 0 (mmHg)	1.01 (0.95–1.08)	**1.08 (1.02–1.15)**
	ptime > 0 (%)	1.05 (0.90–1.24)	**1.24 (1.08–1.44)**

The derived odds ratios are displayed for every 1000 mmHg*h Dose, 10 mmHg hDose, and 5% ptime

The results of the ordinal analyses are shown either after adjustment of the outcome definition depending on the clinical prognosis (i.e. sliding dichotomy) or when assessed across the different GOS thresholds (i.e. proportional odds regression). The metrics for which the bootstrapping derived 95% confidence intervals do not cross 1 are highlighted in bold

*CPP* cerebral perfusion pressure, *CPPopt* optimal CPP, *hDose* hourly dose, *LLA* lower limit of autoregulation, *ULA* upper limit of autoregulation, *ptime* percentage time

^*^Data shown as median (interquartile range)

**Fig. 3:**
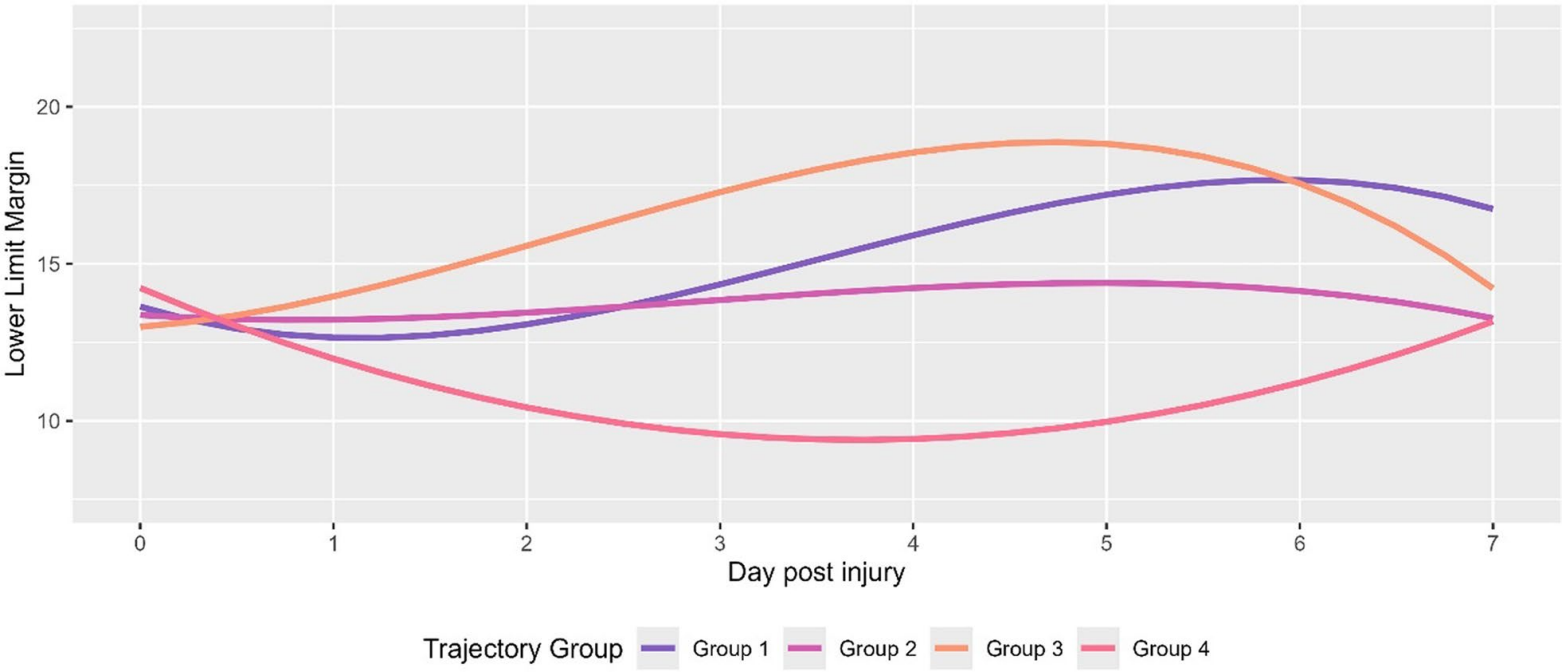
Trajectory Modeling. The different lower limit margin (i.e. deviation between CPPopt and LLA) trajectories identified using the optimal model, which comprised four trajectories (each characterized by cubic polynomials) are displayed as a function of time. The temporal change differs by trajectory with an early increase in the lower limit margin in Group 3 and a slightly later increase in Group 1. In Group 2, the trajectory remains largely stable, while in Group 4 there is a distinct decrease over time

Finally, group-based trajectory modeling was conducted to identify subgroups of patients with similar lower limit margin time trends. The optimal model comprised four trajectories, each characterized by cubic polynomials (Fig. 3). The average posterior probability of membership exceeded 90% for all trajectories. Trajectory 1 exhibited the earliest increase in the lower limit margin, followed by Trajectory 3. In contrast, Trajectory 2 remained stable throughout, whereas Trajectory 4 displayed a marked decrease, dropping to around 6 mmHg between days 2 and 5 post-injury. Statistical analysis (Table 4) indicated that age was the only clinical variable differing among trajectories, with older patients predominantly found in Trajectories 2 and 4 (*p* < 0.001). Regarding outcomes, patients in Trajectories 1 and 3 were significantly more likely to attain a favorable outcome (GOS 4/5) than those in Trajectories 2 and 4 (54%, 32%, 52%, and 28%, respectively; *p* = 0.011). Although the rate of excellent recovery (GOS 5) was comparable in Trajectories 1, 2, and 3, it was notably lower in Trajectory 4 (17%, 19%, 18%, and 3.3%, respectively; *p* = 0.025). Similarly, mortality was highest in Trajectory 4, followed by Trajectory 2, and lowest in Trajectories 1 and 3 (45%, 35%, 23%, and 18%, respectively; *p* = 0.003).

**Table 4:** Patient characteristics stratified by lower limit margin trajectory*

Characteristic	Trajectory				*p*-value
	1 *N* = 35	2 *N* = 31	3 *N* = 85	4 *N* = 60	
Sex (male)	24 (69%)	27 (87%)	68 (80%)	44 (73%)	0.3
Age (years)	35 (25, 55)	55 (35, 65)	45 (25, 55)	55 (35, 65)	< 0.001
Motor GCS	3 (1, 5)	4 (1, 6)	4 (1, 5)	4 (1, 5)	0.6
*Pupillary reactivity*					0.2
Both reactive	21 (60%)	20 (65%)	64 (75%)	38 (63%)	
One reactive	4 (11%)	7 (23%)	10 (12%)	8 (13%)	
None reactive	10 (29%)	4 (13%)	11 (13%)	14 (23%)	
DC	9 (26%)	7 (23%)	23 (27%)	23 (38%)	0.3
Isolated TBI	10 (29%)	11 (35%)	15 (18%)	12 (20%)	0.2
*Intracranial injury*					
EDH	2 (5.7%)	5 (16%)	16 (19%)	7 (12%)	0.3
ICH	8 (23%)	3 (9.7%)	16 (19%)	15 (25%)	0.4
SDH	20 (57%)	23 (74%)	59 (69%)	47 (78%)	0.2
Contusion	18 (51%)	17 (55%)	50 (59%)	40 (67%)	0.5
tSAH	19 (54%)	19 (61%)	60 (71%)	41 (68%)	0.3
*Extracranial injury*					
Abdomen	7 (20%)	3 (9.7%)	11 (13%)	12 (20%)	0.5
Bone extremities	8 (23%)	5 (16%)	20 (24%)	8 (13%)	0.4
Pelvis	5 (14%)	2 (6.5%)	9 (11%)	9 (15%)	0.6
Skull	16 (46%)	13 (42%)	48 (56%)	37 (62%)	0.2
Spine	7 (20%)	7 (23%)	15 (18%)	15 (25%)	0.7
Thorax	16 (46%)	10 (32%)	39 (46%)	27 (45%)	0.6
Outcome—Favorable (GOS 4/5)	19 (54%)	10 (32%)	44 (52%)	17 (28%)	0.011
Outcome—Excellent recovery (GOS 5)	6 (17%)	6 (19%)	15 (18%)	2 (3.3%)	0.025
Outcome—Mortality	8 (23%)	11 (35%)	15 (18%)	27 (45%)	0.003

The clinical parameters depending on trajectory of the deviation between CPPopt and LLA are shown. The corresponding trajectories can be found in Fig. 3

*GCS* glasgow coma scale, *GOS* glasgow outcome scale, *DC* decompressive craniectomy, *EDH* extradural hematoma, *ICH* intracerebral hematoma, *SDH* subdural hematoma, *tSAH* traumatic subarachnoid hemorrhage, *TBI* traumatic brain injury

^*^Data shown as median (interquartile range) or number (%)

## Discussion

Drawing on experimental insights from animal studies, we examined both the direct relationship between various CPP levels and PRx, and the prognostic value of different personalized, autoregulation-based CPP targets in a cohort of over 800 consecutive TBI patients. The key findings were:

1. Across potential confounders such as age, outcome, and ICP level, the CPP/PRx curve consistently exhibited an asymmetric pattern with a more pronounced deterioration of cerebrovascular reactivity with decreasing compared to increasing CPP.
2. Deviations below the LLA had the strongest association with worse outcomes, and similarly, falling below CPPopt was associated with unfavorable outcomes. Conversely, deviations above CPPopt seemed to be generally linked with improved outcomes. However, this association was overall weaker and, coupled with prolonged exposure time of values well above ULA, it inversed its character, showing a weak, and somewhat inconsistent, association with worse outcome.
3. The relationship between CPPopt and LLA evolved over time. Different lower limit margin trajectories could be identified, each with a distinct pattern of outcomes.

Our results align with the experimental findings described by Klein et al. [12] as well as early work by Aries et al. [7]. Specifically, our data demonstrates that the increase in passive reactivity with rising CPP is less steep compared to its increase with decreasing CPP in an adult cohort of TBI patients. As CPP decreases below the LLA, the relaxation of the smooth muscle cells, a passive process, becomes increasingly insufficient for maintaining cerebral perfusion with passive narrowing of the vasculature [13]. Conversely, above the ULA, increases in CPP are still, for a certain CPP range, counteracted by active smooth muscle cell contraction, insufficient however at this point to completely keep the blood flow constant [12]. Additional nuances were apparent when examining different subgroups. With advancing age, there appears to be a decline in autoregulatory efficiency, as evidenced by higher PRx values at the optimal CPP. This may be attributed to the impaired function of smooth muscle cells associated with age-related conditions (e.g. arteriosclerosis, vessel wall fibrosis, and inflammation—common sequelae of chronic hypertension and type 2 diabetes) [32–34]. Indeed, previous research [35] indicated that older patients exhibit diminished cerebrovascular reactivity and may not benefit from adjusting CPP to the optimal value, given that reactivity remains suboptimal even at CPPopt. In contrast, younger patients often exhibit more robust autoregulatory function, with the CPP/PRx relationship typically presenting only a lower limit without a defined upper limit [36].

Metrics derived from personalized CPP targets consistently outperformed the fixed targets recommended by the Brain Trauma Foundation. In agreement with earlier studies [8, 9, 37], we found that deviations below CPPopt and LLA were strongly and independently associated with worse outcomes. However, no association emerged between greater time spent above CPPopt and adverse outcomes. In contrast, patients in our cohort who had higher ptime and hDose above CPPopt were less likely to die. Our findings consolidate previous findings which identified an association between CPP deviations above CPPopt and improved long-term recovery potential [38]. Furthermore, they align with detailed physiological analyses demonstrating that modest increases in CPP—approximately 10 mmHg above CPPopt—are accompanied by an improvement of in cerebral physiology including reduced ICP and an increased partial pressure of brain tissue oxygen [39]. Only above the ULA and with more sensitive analyses, were we able to identify a negative association between high CPP beyond the ULA and worse outcomes. Several factors could explain the lack of an association between worse outcomes and elevated CPP above CPPopt. First, the observed asymmetry in CPP/PRx relationship suggests that autoregulation is more tolerant of increasing than decreasing CPP. Additionally, cerebral oxygenation—essential for meeting cerebral metabolic demands—declines with decreasing CPP, while remaining relatively stable at higher CPP levels, until critically elevated values are reached, particularly in patients with impaired autoregulation [40, 41].

Importantly, to date, the region surrounding CPPopt entailing CPP values from 5 mmHg below to 5 mmHg above CPPopt has arbitrarily been used to define the optimal target for management [42, 43] and prognostication [37, 38]. Our findings indicate that it may be more appropriate to consider CPPopt as the lower boundary of the safe CPP range rather than a precise optimal target. Notably, due to the nature of PRx, partially impaired cerebrovascular reactivity (as is the case below CPPopt) may be reflective of regional heterogeneity in autoregulatory capacity, with some brain regions being more affected than others, potentially already contributing to worse outcomes. This suggests that even if PRx is still below the conventional threshold of 0.3, a trend toward worsening PRx with decreasing CPP likely indicates that some regions have already fallen below the LLA. In contrast, a markedly elevated PRx more likely reflects a global failure of autoregulatory mechanisms. The importance of preserving adequate distance from the LLA is underscored by the distinct trajectories in the lower limit margin identified in our study: the smaller the margin between CPPopt and the LLA, the higher the likelihood of an unfavorable outcome. In the trajectory associated with the worst outcomes, this distance dropped to below 10 mmHg, highlighting the critical need to maintain an adequate safety margin.

### Limitations

A key limitation of this study is the heterogeneity between the two cohorts included. Notably, patients in the more recent cohort were older, more frequently presented with non-reactive pupils, and had lower rates of favorable outcome, which may influence the interpretation and generalizability of the findings. The TBI 2002–2020 cohort was acquired through unsystematic data collection over a long period during which management strategies evolved [16], potentially introducing variability in patient selection and treatment. In contrast, the TBI 2021–2023 cohort was enrolled systematically [18], better reflecting contemporary clinical practices. However, secondary analyses restricted to the TBI 2021–2023 cohort yielded consistent results, supporting the robustness of our main conclusions. One additional key limitation involves the calibration of the arterial pressure transducer. Before 2015, transducers were zeroed at the level of the right atrium, whereas from 2015 onward, they were zeroed approximately at the level of the foramen of Monro (as estimated by tragus level). Based on the management regimen used in our institution [15] which includes 30° head elevation and based on the thereby generated change in hydrostatic pressure [20], we estimated an offset of 10 mmHg. While this estimate likely approximates the actual CPP zeroed at the foramen of Monro, during changes in head elevation (e.g. for CT-scans) or dependent on the patient-specific distance between the foramen of Monro and the right atrium, the offset might differ. Of note, this difference in zeroing does not affect the calculation of PRx [44]. Similarly, the derived CPP targets (i.e. CPPopt, LLA, ULA) are independent of the zeroing since they are patient specific. However, to account for these discrepancies, the multivariable analyses were restricted to patients whose arterial pressure transducers were zeroed at the foramen of Monro. Several other relevant changes in treatment practice occurred during this period, including the introduction of bedside monitoring of CPPopt in 2012 [16]. Of note, while PRx may be improved by adjusting CPP targets under certain circumstances [17], currently no other intervention can alter the underlying physiological relationship between CPP and PRx. Because our cohort was observational, all patients received active treatment, with a subset receiving PRx optimization via CPPopt based CPP management [43]. While our results support the detrimental effect of CPP below personalized limits on outcome, this study cannot prove the safety of CPP values beyond and above CPPopt. Current guidelines advise caution against aggressive maintenance of CPP above 70 mmHg to avoid complications such as respiratory failure [1, 45]. However, robust evidence is still lacking, and further investigations are critical in view of evolving ventilation strategies [46, 47] and recent data suggesting no clear association between increased CPP and the incidence of acute respiratory distress syndrome [48]. Lastly, a major limitation lies in the automated estimation of personalized CPP targets. The methods employed assume a symmetrical relationship between CPP and PRx, which is not always the case. Furthermore, the full curve is fitted even when the upper limit is not reached, thereby potentially introducing inaccuracies in the estimation process [10].

## Conclusion

Personalized CPP targets consistently outperform fixed thresholds for prognostication after TBI. Our findings challenge the assumption that maintaining CPP within ± 5 mmHg of CPPopt is optimal. Even slight deviations below CPPopt were linked to worse outcomes, especially in patients with a narrow lower limit margin. Clinically, this supports using CPPopt as the lower CPP threshold, as deviations above it were not associated with poorer, but at times even better outcomes. This disparity is likely attributable to the asymmetry in the CPP/PRx relationship and different mechanisms of secondary injury at the two extremes of CPP.

## Supplementary Information

Supplement1. Strobe Statement

Supplement2. Supplement A-E

## Data Availability

Postprocessed data is available upon reasonable request to the corresponding author.
