# Supplementary material for: Cerebral perfusion pressure targets after traumatic brain injury: a reappraisal
Source: Crit Care. 2025 May 21;29:207. doi: 10.1186/s13054-025-05458-9 (PMC12096506; doi:10.1186/s13054-025-05458-9)

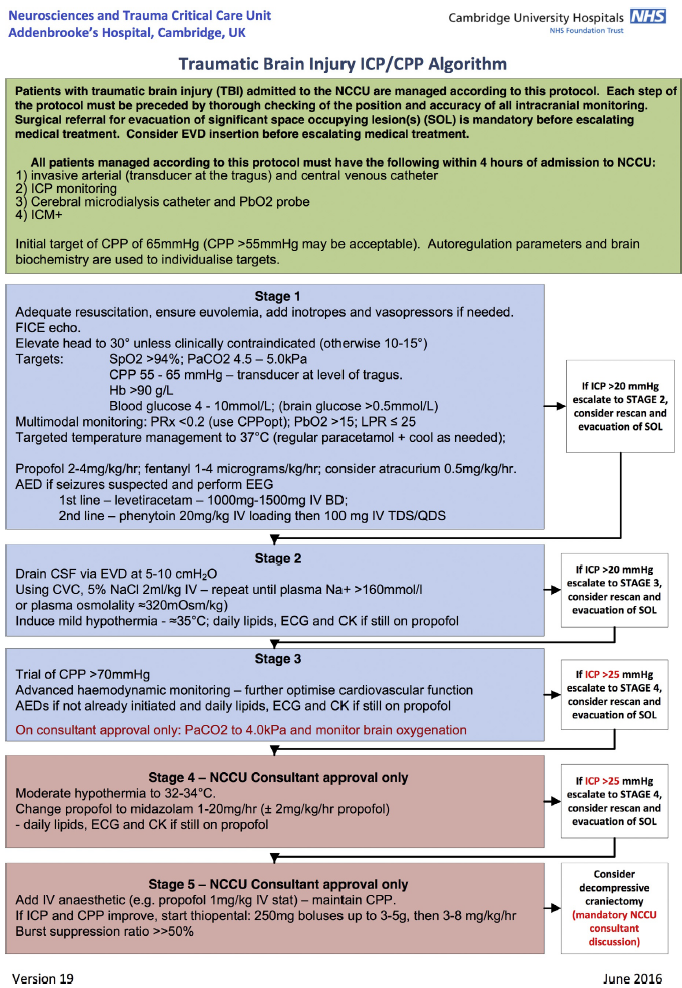
**Supplement A: TBI ICP/CPP Management Algorithm.** Local intracranial pressure and cerebral perfusion pressure management protocol. The specific treatment protocol has been described previously (Menon D, Ercole A. Critical care management of traumatic brain injury. Handbook of clinical neurology. 2017; 140:239-74).

**Supplement B: R Packages used.** The specific R packages used for the presented analysis are: *dplyr, rstatix, gtsummary, MatchIt, optmatch, cobalt, MASS, gbmt, ggplot2.*

**Supplement C: CPP Data Coverage.** The CPP data coverage is shown in form of boxplots (length – A; stratified by outcome GOS 1-3 vs. 4-5) and density plots (frequency of data relative to day post injury or onset of monitoring (with day 0 being the day of the initial injury for the cohort TBI 2021-2023 and with day 0 being the start of recording for the cohort TBI 2002-2020) - B; stratified by GOS category. The median number of hours available was 163 (IQR 75-249) hours for CPP. No difference in length of stay could be identified when considering patients with unfavorable vs. favorable outcomes (i.e. GOS 1-3 vs. GOS 4/5) with median 167 (IQR 94-297) vs. 150 (IQR 83-279) and 113 (IQR 53-195) vs. 110 (IQR 44-191) for the TBI 2002-2020 and TBI 2021-2023 cohorts respectively (p= 0.4 and 0.3 respectively). The vast majority was acquired within the first week of injury with similar distributions throughout the hospital stay irrespective of GOS category.


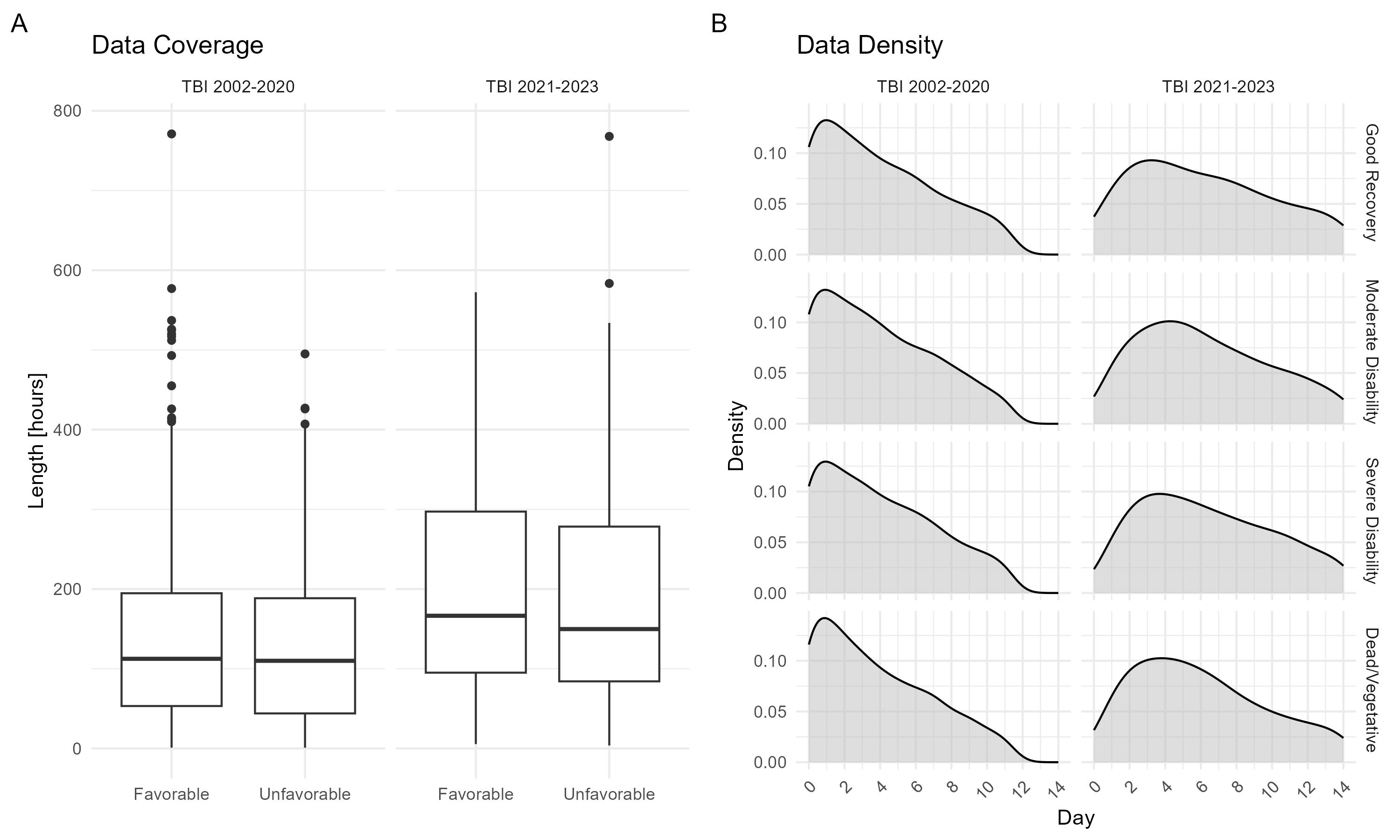


**Supplement D: Results of Logistic Regression Analyses.** The results of the iterative logistic regression analyses are shown using odds ratios (OR) and confidence intervals (95% CI) for the different absolute CPP cutoffs (top table) and the personalized CPP targets. The derived odds ratios are displayed for every 1000 mmHg*h Dose, 10 mmHg hDose, and 5% ptime. For the personalized CPP targets, the number behind the target represents the deviation from the cutoff (e.g. LLA 5 uses the cutoff that is 5 mmHg below the LLA).

| **CPP** | **Dose** | | **hDose** | | **ptime** | |
| --- | --- | --- | --- | --- | --- | --- |
|  | **OR (95% CI)** | **p-value** | **OR (95% CI)** | **p-value** | **OR (95% CI)** | **p-value** |
| <30 mmHg | 1.01 (0.99 - 1.04) | 0.491 | 1.03 (1.00 - 1.08) | 0.069 | 1.39 (1.08 - 2.11) | **0.044** |
| <35 mmHg | 1.01 (0.99 - 1.03) | 0.471 | 1.03 (1.01 - 1.07) | 0.053 | 1.42 (1.11 - 2.15) | **0.035** |
| <40 mmHg | 1.01 (0.99 - 1.03) | 0.425 | 1.03 (1.01 - 1.07) | **0.038** | 1.51 (1.15 - 2.34) | **0.020** |
| <45 mmHg | 1.01 (0.99 - 1.03) | 0.362 | 1.03 (1.01 - 1.06) | **0.029** | 1.64 (1.22 - 2.61) | **0.009** |
| <50 mmHg | 1.01 (0.99 - 1.03) | 0.286 | 1.03 (1.01 - 1.06) | **0.023** | 1.69 (1.29 - 2.47) | **0.002** |
| <55 mmHg | 1.01 (1.00 - 1.03) | 0.195 | 1.03 (1.01 - 1.06) | **0.017** | 1.48 (1.25 - 1.84) | **<0.001** |
| <60 mmHg | 1.01 (1.00 - 1.03) | 0.100 | 1.03 (1.01 - 1.07) | **0.007** | 1.24 (1.14 - 1.37) | **<0.001** |
| <65 mmHg | 1.01 (1.00 - 1.02) | **0.039** | 1.03 (1.02 - 1.05) | **0.001** | 1.11 (1.05 - 1.16) | **<0.001** |
| >65 mmHg | 1.00 (1.00 - 1.00) | **0.026** | 1.00 (0.99 - 1.00) | 0.213 | 0.90 (0.86 - 0.95) | **<0.001** |
| >70 mmHg | 1.00 (1.00 - 1.00) | **0.031** | 1.00 (0.99 - 1.00) | 0.415 | 0.95 (0.92 - 0.98) | **0.003** |
| >75 mmHg | 1.00 (0.99 - 1.00) | **0.042** | 1.00 (0.99 - 1.00) | 0.636 | 0.98 (0.95 - 1.01) | 0.123 |
| >80 mmHg | 1.00 (0.99 - 1.00) | 0.057 | 1.00 (0.99 - 1.01) | 0.799 | 0.99 (0.96 - 1.02) | 0.386 |
| >85 mmHg | 1.00 (0.99 - 1.00) | 0.071 | 1.00 (0.99 - 1.01) | 0.920 | 0.99 (0.96 - 1.03) | 0.585 |
| >90 mmHg | 0.99 (0.98 - 1.00) | 0.087 | 1.00 (0.99 - 1.01) | 0.944 | 0.99 (0.95 - 1.03) | 0.664 |
| >95 mmHg | 0.99 (0.98 - 1.00) | 0.109 | 1.00 (0.99 - 1.02) | 0.817 | 1.00 (0.94 - 1.06) | 0.917 |
| >100 mmHg | 0.98 (0.96 - 1.00) | 0.128 | 1.00 (0.98 - 1.03) | 0.703 | 1.00 (0.93 - 1.08) | 0.993 |
| >105 mmHg | 0.97 (0.93 - 1.00) | 0.140 | 1.01 (0.98 - 1.05) | 0.628 | 1.01 (0.91 - 1.14) | 0.819 |
| >110 mmHg | 0.95 (0.88 - 1.01) | 0.161 | 1.01 (0.96 - 1.08) | 0.587 | 1.04 (0.88 - 1.25) | 0.625 |
| >115 mmHg | 0.92 (0.79 - 1.01) | 0.160 | 1.02 (0.94 - 1.13) | 0.617 | 1.08 (0.84 - 1.44) | 0.547 |
| >120 mmHg | 0.85 (0.66 - 1.02) | 0.140 | 1.03 (0.91 - 1.20) | 0.677 | 1.12 (0.78 - 1.79) | 0.558 |

*Part 1*

*Part 2*

| **Variable** | **Dose** | | **hDose** | | **ptime** | |
| --- | --- | --- | --- | --- | --- | --- |
|  | **OR (95% CI)** | **p-value** | **OR (95% CI)** | **p-value** | **OR (95% CI)** | **p-value** |
| < LLA 25 | 2.06 (1.21 - 3.97) | **0.017** | 2.26 (1.39 - 4.32) | **0.005** | 11.13 (2.93 - 15.00) | **0.003** |
| < LLA 20 | 1.72 (1.25 - 2.51) | **0.002** | 1.95 (1.38 - 3.04) | **0.001** | 9.15 (3.73 - 15.00) | **<0.001** |
| < LLA 15 | 1.44 (1.20 - 1.75) | **<0.001** | 1.72 (1.38 - 2.23) | **<0.001** | 4.29 (2.65 - 7.48) | **<0.001** |
| < LLA 10 | 1.23 (1.12 - 1.36) | **<0.001** | 1.41 (1.25 - 1.60) | **<0.001** | 2.02 (1.62 - 2.62) | **<0.001** |
| < LLA 5 | 1.11 (1.06 - 1.17) | **<0.001** | 1.20 (1.14 - 1.28) | **<0.001** | 1.46 (1.30 - 1.66) | **<0.001** |
| < LLA | 1.05 (1.03 - 1.08) | **<0.001** | 1.11 (1.07 - 1.14) | **<0.001** | 1.26 (1.18 - 1.35) | **<0.001** |
| < CPPopt 25 | 1.14 (1.02 - 1.31) | **0.040** | 1.40 (1.17 - 1.73) | **0.001** | 2.48 (1.62 - 4.02) | **<0.001** |
| < CPPopt 20 | 1.08 (1.01 - 1.16) | **0.023** | 1.24 (1.12 - 1.39) | **<0.001** | 1.60 (1.29 - 2.03) | **<0.001** |
| < CPPopt 15 | 1.04 (1.01 - 1.08) | **0.029** | 1.13 (1.07 - 1.19) | **<0.001** | 1.28 (1.14 - 1.46) | **<0.001** |
| < CPPopt 10 | 1.02 (1.00 - 1.04) | 0.065 | 1.07 (1.04 - 1.10) | **<0.001** | 1.14 (1.07 - 1.23) | **<0.001** |
| < CPPopt 5 | 1.01 (1.00 - 1.02) | 0.193 | 1.04 (1.02 - 1.06) | **<0.001** | 1.09 (1.04 - 1.15) | **0.001** |
| < CPPopt | 1.00 (1.00 - 1.01) | 0.507 | 1.03 (1.01 - 1.04) | **<0.001** | 1.07 (1.02 - 1.12) | **0.009** |
| > CPPopt | 0.99 (0.99 - 1.00) | **0.017** | 0.99 (0.98 - 1.00) | 0.157 | 0.94 (0.89 - 0.98) | **0.009** |
| > CPPopt 5 | 0.99 (0.98 - 1.00) | **0.028** | 0.99 (0.98 - 1.01) | 0.349 | 0.95 (0.90 - 1.00) | 0.072 |
| > CPPopt 10 | 0.99 (0.97 - 1.00) | **0.045** | 0.99 (0.97 - 1.01) | 0.479 | 0.97 (0.91 - 1.05) | 0.461 |
| > CPPopt 15 | 0.98 (0.96 - 1.00) | 0.066 | 0.99 (0.96 - 1.02) | 0.545 | 0.96 (0.87 - 1.06) | 0.440 |
| > CPPopt 20 | 0.97 (0.93 - 1.01) | 0.113 | 0.99 (0.95 - 1.03) | 0.592 | 0.97 (0.84 - 1.11) | 0.629 |
| > CPPopt 25 | 0.96 (0.89 - 1.02) | 0.172 | 0.98 (0.91 - 1.04) | 0.502 | 0.99 (0.82 - 1.20) | 0.932 |
| > ULA | 1.00 (0.98 - 1.02) | 0.820 | 1.03 (1.01 - 1.06) | **0.018** | 1.11 (1.04 - 1.18) | 0.002 |
| > ULA 5 | 1.01 (0.97 - 1.05) | 0.558 | 1.04 (1.00 - 1.08) | 0.084 | 1.17 (1.05 - 1.31) | 0.006 |
| > ULA 10 | 1.03 (0.97 - 1.11) | 0.342 | 1.04 (0.98 - 1.11) | 0.205 | 1.17 (0.98 - 1.41) | 0.085 |
| > ULA 15 | 1.09 (0.96 - 1.25) | 0.193 | 1.06 (0.96 - 1.17) | 0.286 | 1.22 (0.93 - 1.65) | 0.168 |
| > ULA 20 | 1.20 (0.97 - 1.54) | 0.127 | 1.07 (0.91 - 1.27) | 0.432 | 1.39 (0.90 - 2.29) | 0.157 |
| > ULA 25 | 1.35 (0.94 - 2.11) | 0.136 | 1.04 (0.82 - 1.36) | 0.738 | 1.68 (0.79 - 3.97) | 0.198 |


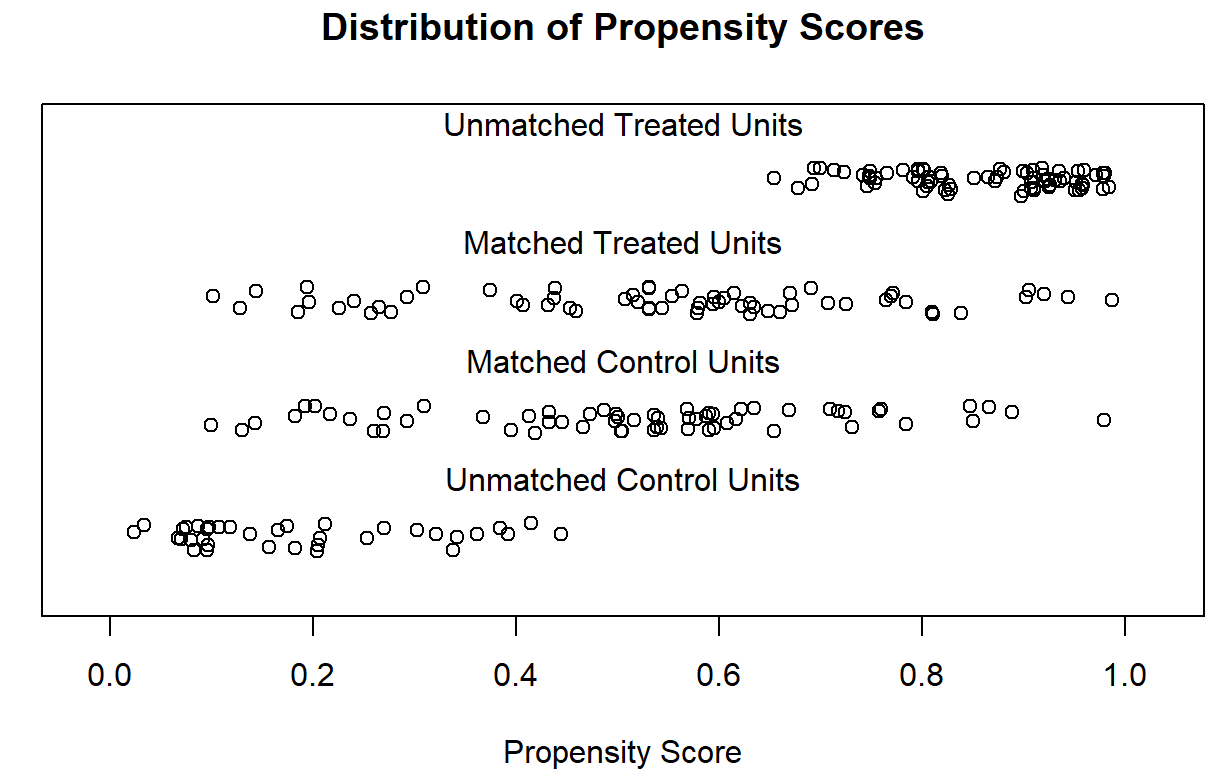

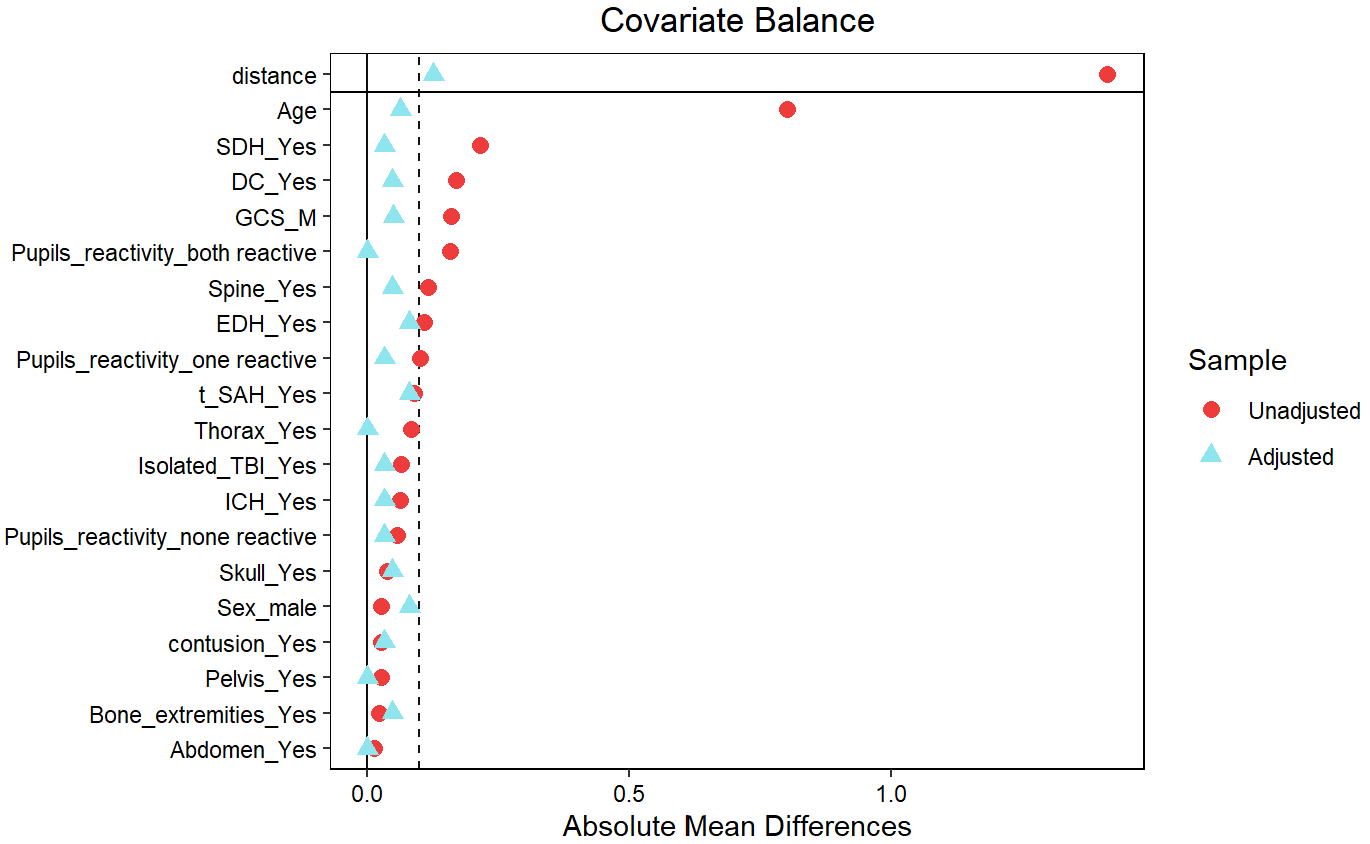

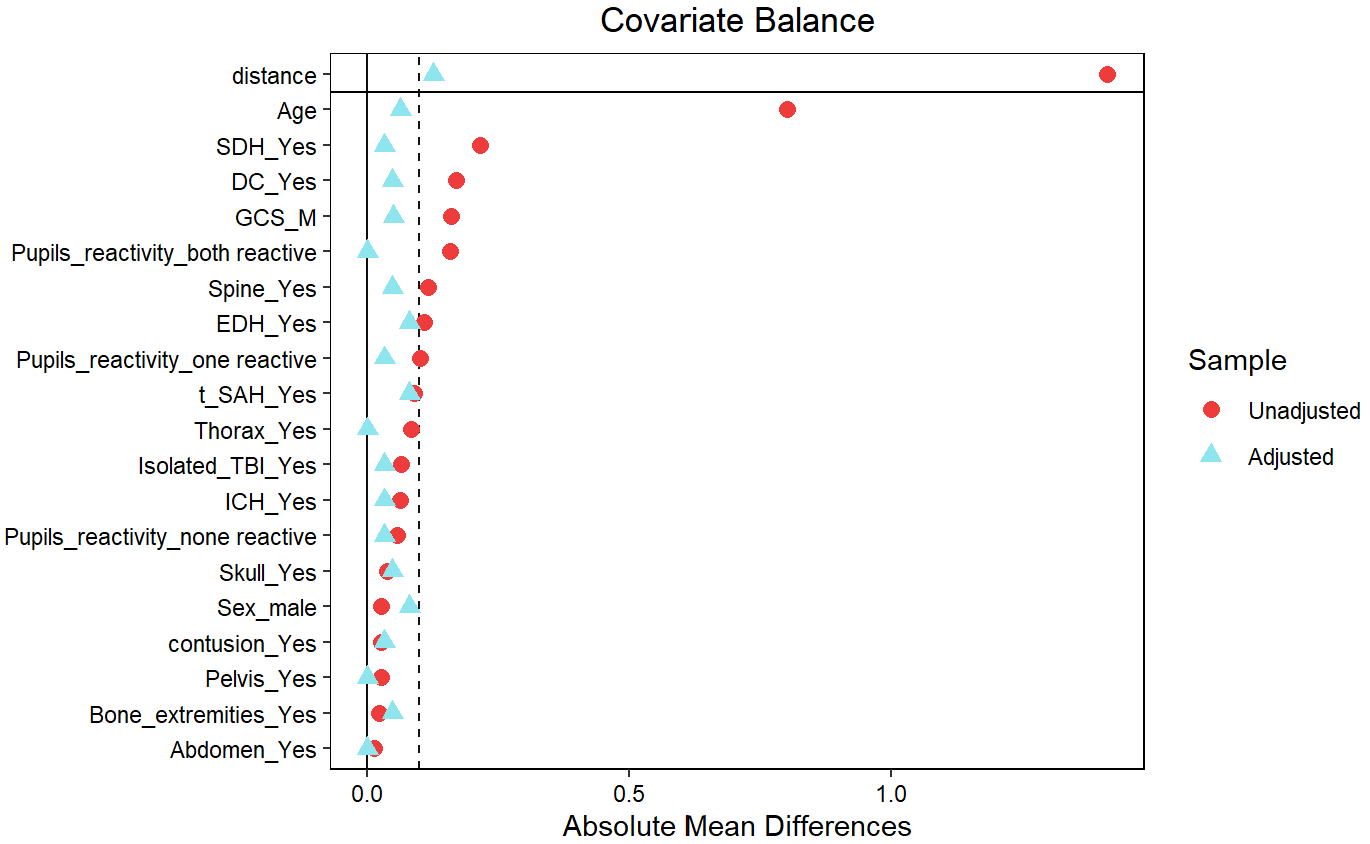

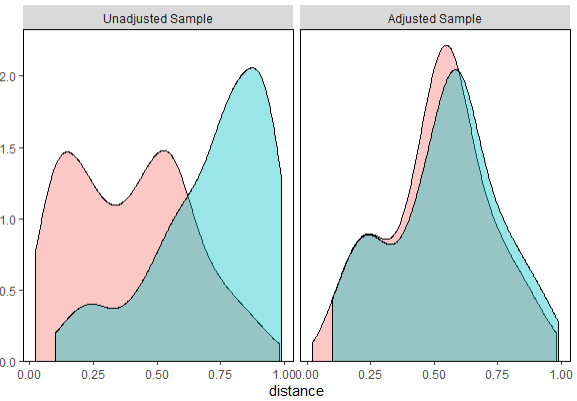
**Supplement E: Matching based on prognostic risk scores.** Matching based on prognostic risk scores was performed using the nearest-neighbor method with a caliper of 0.2 and 1:1 matching to ensure that matches were within a reasonable distance in terms of their risk scores. The prognostic risk scores were built using logistic regression comparing favorable vs. unfavorable outcome. The results of the matching procedure are shown using density distribution of scores before and after adjustment (blue: favorable outcome, red: unfavorable outcome), point distributions of matched and unmatched units, and covariate balance before and after matching. In addition, the variables (pre and post matching) are shown in the table below.

Unmatched: Favorable Outcome

Matched: Favorable Outcome

Matched: Unfavorable Outcome

Unmatched: Unfavorable Outcome


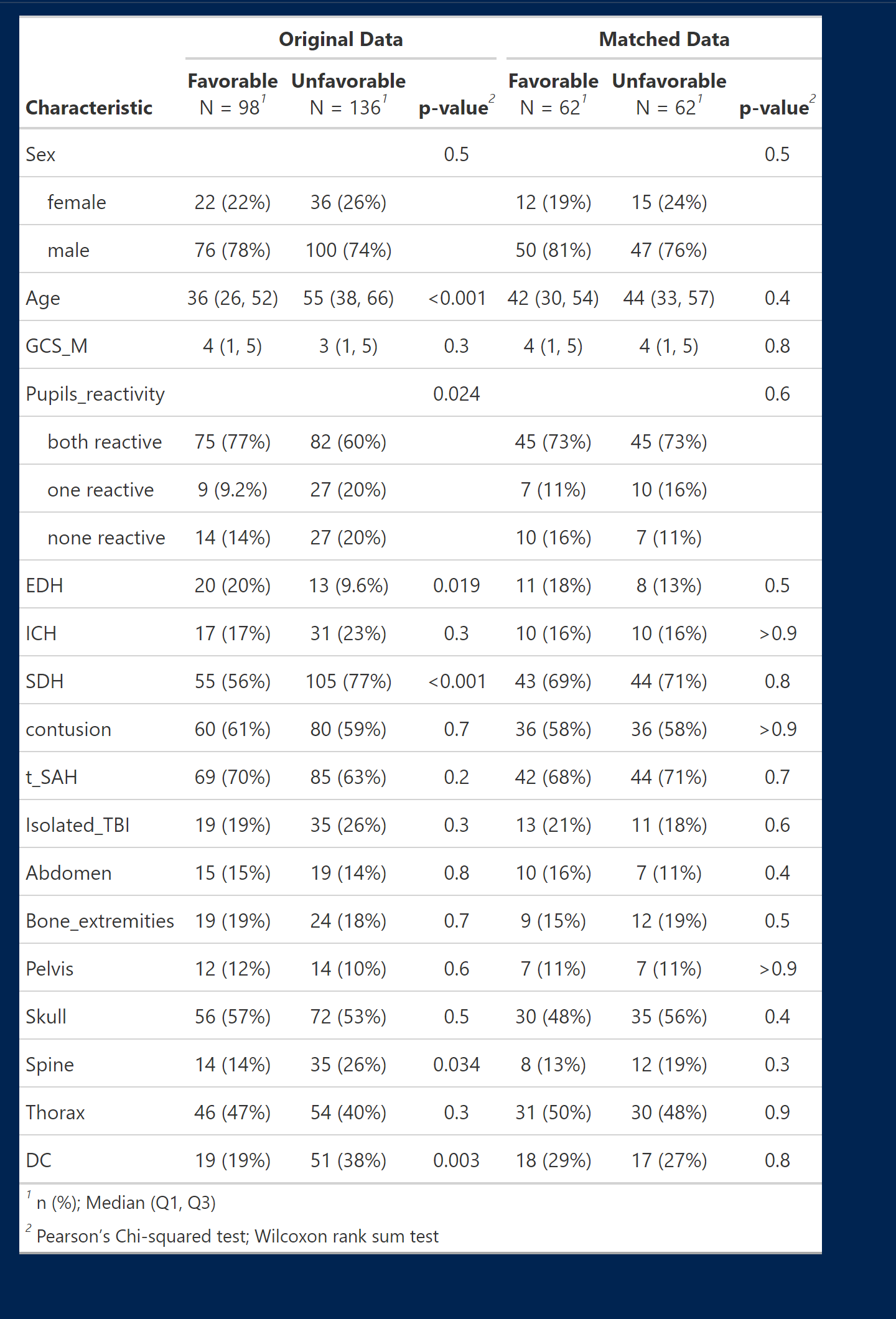

Supplement: Supplementary file 2 — Supplement2. Supplement A-E [file 13054_2025_5458_MOESM2_ESM.docx]
